# Muscular myostatin gene expression and plasma concentrations are decreased in critically ill patients

**DOI:** 10.1186/s13054-022-04101-1

**Published:** 2022-08-03

**Authors:** Julius J. Grunow, Katja Reiher, Niklas M. Carbon, Lilian Jo Engelhardt, Knut Mai, Susanne Koch, Joerg C. Schefold, Werner Z’Graggen, Stefan J. Schaller, Jens Fielitz, Joachim Spranger, Steffen Weber-Carstens, Tobias Wollersheim

**Affiliations:** 1 Department of Anesthesiology and Operative Intensive Care Medicine (CCM/CVK), Charité – Universitätsmedizin Berlin, Corporate Member of Freie Universität Berlin, Humboldt-Universität zu Berlin, Augustenburger Platz 1, 13357 Berlin, Germany; 2 Institute of Medical Informatics, Charité – Universitätsmedizin Berlin, Corporate Member of Freie Universität Berlin, Humboldt-Universität zu Berlin, Charitéplatz 1, 10117 Berlin, Germany; 3 Department of Endocrinology and Metabolic Diseases, Charité – Universitätsmedizin Berlin, Corporate Member of Freie Universität Berlin, Humboldt-Universität Zu Berlin, Charitéplatz 1, 10117 Berlin, Germany; 4 Department of Intensive Care Medicine, Inselspital, Bern University Hospital, University Bern, Freiburgstrasse 18, 3010 Bern, Switzerland; 5 Departments of Neurology and Neurosurgery, Inselspital, Bern University Hospital, University Bern, Freiburgstrasse 18, 3010 Bern, Switzerland; 6 Department of Anesthesiology and Intensive Care, School of Medicine, Technical University of Munich, Ismaninger Straße 22, 81675 Munich, Germany; 7 Charité – Universitätsmedizin Berlin, Max Delbrück Center (MDC) for Molecular Medicine in the Helmholtz Association, Experimental and Clinical Research Center (ECRC), Lindenberger Weg 80, 13125 Berlin, Germany; 8 Department of Internal Medicine B, Cardiology, University Medicine Greifswald, Greifswald, Germany; 9 DZHK (German Center for Cardiovascular Research), Partner Site Greifswald, Greifswald, Germany; 10 Department of Anesthesiology and Operative Intensive Care Medicine (CCM, CVK), Charité - Universitätsmedizin Berlin, Augustenburger Platz 1, 13353 Berlin, Germany

**Keywords:** Myostatin, ICUAW, Muscle atrophy, Insulin resistance, Critical illness

## Abstract

**Background:**

The objective was to investigate the role of gene expression and plasma levels of the muscular protein myostatin in intensive care unit-acquired weakness (ICUAW). This was performed to evaluate a potential clinical and/or pathophysiological rationale of therapeutic myostatin inhibition.

**Methods:**

A retrospective analysis from pooled data of two prospective studies to assess the dynamics of myostatin plasma concentrations (day 4, 8 and 14) and myostatin gene (*MSTN*) expression levels in skeletal muscle (day 15) was performed. Associations of myostatin to clinical and electrophysiological outcomes, muscular metabolism and muscular atrophy pathways were investigated.

**Results:**

*MSTN* gene expression (median [IQR] fold change: 1.00 [0.68–1.54] vs. 0.26 [0.11–0.80]; *p* = 0.004) and myostatin plasma concentrations were significantly reduced in all critically ill patients when compared to healthy controls. In critically ill patients, myostatin plasma concentrations increased over time (median [IQR] fold change: day 4: 0.13 [0.08/0.21] vs. day 8: 0.23 [0.10/0.43] vs. day 14: 0.40 [0.26/0.61]; *p* < 0.001). Patients with ICUAW versus without ICUAW showed significantly lower *MSTN* gene expression levels (median [IQR] fold change: 0.17 [0.10/0.33] and 0.51 [0.20/0.86]; *p* = 0.047). Myostatin levels were directly correlated with muscle strength (correlation coefficient 0.339; *p* = 0.020) and insulin sensitivity index (correlation coefficient 0.357; *p* = 0.015). No association was observed between myostatin plasma concentrations as well as *MSTN* expression levels and levels of mobilization, electrophysiological variables, or markers of atrophy pathways.

**Conclusion:**

Muscular gene expression and systemic protein levels of myostatin are downregulated during critical illness. The previously proposed therapeutic inhibition of myostatin does therefore not seem to have a pathophysiological rationale to improve muscle quality in critically ill patients.

*Trial registration*: ISRCTN77569430—13th of February 2008 and ISRCTN19392591 17th of February 2011.

**Graphical abstract:**

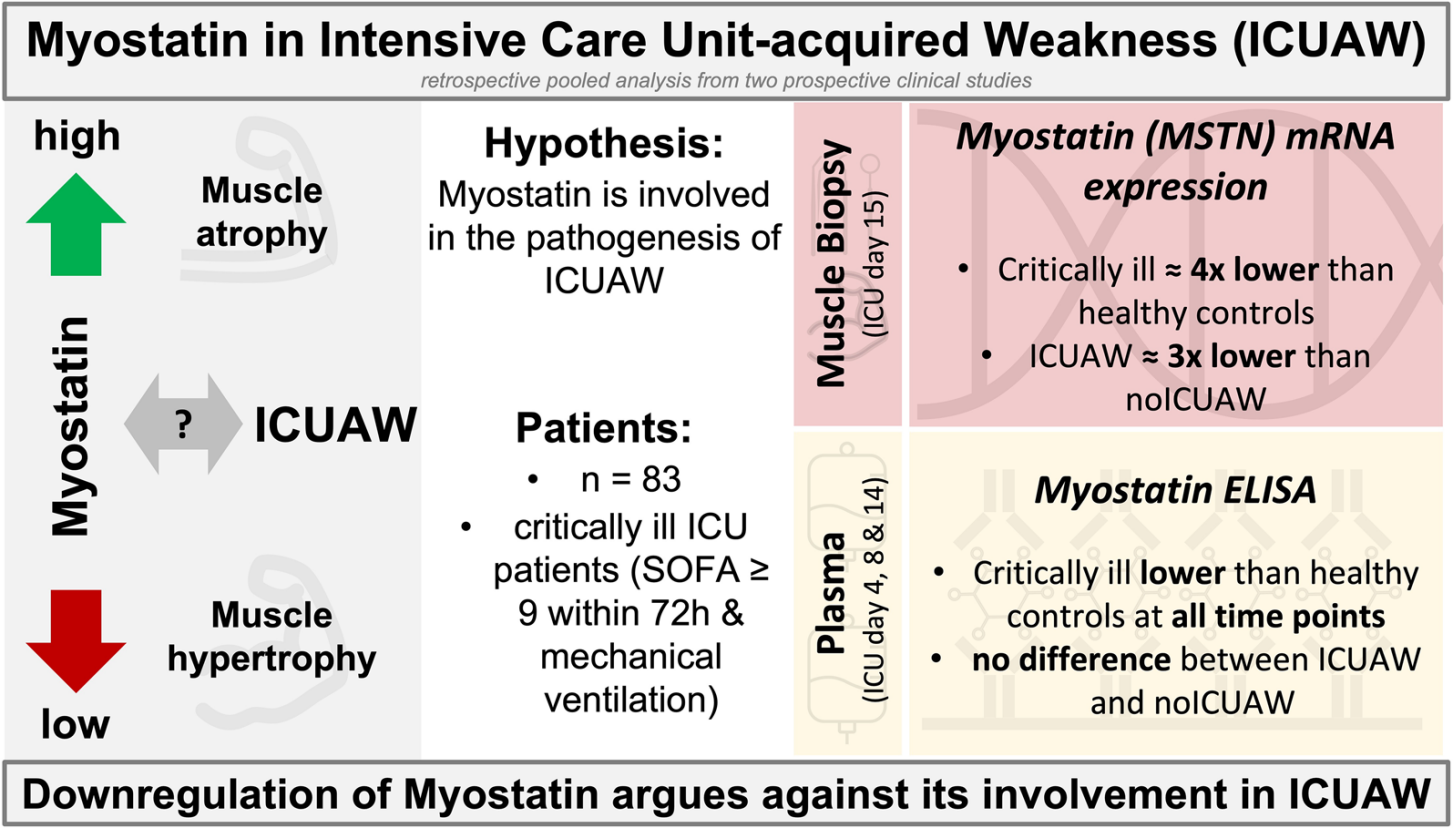

**Supplementary Information:**

The online version contains supplementary material available at 10.1186/s13054-022-04101-1.

## Background

Intensive care unit-acquired weakness (ICUAW) is defined as a clinically relevant muscular weakness with critical illness itself as the most plausible etiology developing in at least 40% of ICU patients [1–3]. ICUAW can be classified in critical illness myopathy (CIM) and/or critical illness polyneuropathy (CIP) and has detrimental effects on weaning from mechanical ventilation, length of hospital stay and ICU mortality as well as long-term outcomes (e.g., physical function, health-related quality of life and survival) [3–5]. Severe muscle atrophy with a muscle mass decrease of 4% per day accompanies the described ICUAW [6–8]. Even though a latter increase in muscle mass (6-month post-ICU discharge) has been described, atrophy is still evident in most patients [9]. The ubiquitin–proteasome system as a key pathway of muscle protein degradation is activated early during critical illness. Simultaneously, a reduction in muscle protein synthesis measured via myostatin heavy chain expression can be observed [3, 6], whereas incorporation of labeled amino acid does not show a decrease [10]. Besides, muscle atrophy is insulin resistance also commonly present during critical illness and especially pronounced in patients with neuromuscular dysfunction [11]. It has been shown that overcoming insulin resistance via intensive insulin therapy has a protective effect for the neuromuscular function in critically ill patients [12, 13]. While these mechanisms contribute largely to the clinical weakness, the definitive pathophysiology of ICUAW remains under investigation.

Myostatin (GDF-8) is a growth and differentiating factor involved in muscle mass regulation during embryonic development and plays a key role in (patho-)physiologic adaptations of skeletal muscle mass [14]. Loss of myostatin function leads to skeletal muscle hypertrophy, hyperplasia and finally a massively increased muscle mass in humans, mice and cattle, with myostatin being highly conserved across species [14–17]. In contrast, myostatin overexpression has been shown to be involved in muscle atrophy during different diseases such as chronic heart failure or chronic obstructive pulmonary disease [18, 19]. Myostatin mediates atrophy in a Forkhead box O 1 (FoxO1)-dependent manner in vivo and in vitro via upregulation of Atrogin-1 and muscle RING-finger protein-1 (MuRF1) and suppresses muscle protein synthesis reflected by a reduction in myosin heavy chain expression, which is also observed during ICUAW [6, 20]. Myostatin has also been shown to play a role in insulin resistance as it inversely correlates with insulin sensitivity in healthy adults [21, 22]. Furthermore, inhibition of myostatin in murine models has led to improved insulin sensitivity and increased GLUT4 expression, which are both impaired in critically ill patients [11, 23, 24]. Nevertheless, it remains unclear to what extent myostatin is involved in the regulation of muscle atrophy as well as insulin resistance during ICUAW.

Aerobic and resistance exercise have been shown to decrease myostatin levels and improve insulin sensitivity in healthy human subjects [22, 25]. Evidence suggests that this could also be the case in critically ill patients as muscle activation has been shown to restore impaired GLUT4 translocation and prevent muscle atrophy [11, 26]. Early mobilization is generally recommended for critically ill patients due to its beneficial clinical effects [27, 28]. Whether myostatin is involved in the beneficial effects of early mobilization in critically ill patients has not been investigated so far.

Pharmaceutical inhibition of myostatin is possible and has been investigated in different diseases (NCT02310763, NCT02907619). Investigations in critically ill patients have not been conducted as the role of myostatin during critical illness is unknown.

We hypothesized that myostatin mRNA expression and systemic protein levels are elevated during the early and acute phase of critical illness in patients with ICUAW and that this is associated with muscle atrophy, development of CIP and CIM and insulin resistance. We further hypothesized that the beneficial effects of early mobilization are due to a suppression of myostatin rendering pharmaceutical inhibition as a possible prophylactic or therapeutic intervention to improve muscle quality.

## Material and methods

### Study design

We performed a retrospective analysis in a pooled adult patient cohort that participated in two prospective clinical studies (ISRCTN77569430 [6] and ISRCTN19392591 [26]) conducted in the same ICUs at a tertiary university care center (Charité – Universitätsmedizin Berlin). The first study was an observational study investigating pathomechanisms of ICUAW, while the second study was a randomized controlled interventional trial investigating the effects of protocol-based physiotherapy with and without early muscle activating measures on ICUAW. Patients were enrolled following written informed consent provided by a legal proxy. Ethical approval for both studies had been granted by the institutional review board (EA2/061/06 and EA2/041/10) and conformed to the Declaration of Helsinki.

### Participants

Patients in both studies fulfilled the same in- and exclusion criteria. The following criteria were necessary for inclusion: age ≥ 18 years, mechanical ventilation, and high risk for developing ICUAW (defined as SOFA score ≥ 9 within the first 72 h after ICU admission). Exclusion criteria encompassed: Body Mass Index > 35 kg/m^2^, futile prognosis with high likelihood of death in the following hours, previously known neuromuscular disease and/or insulin-dependent diabetes mellitus. For reference values from healthy individuals, muscle biopsy specimens from 11 and plasma samples from 91 healthy volunteers were used. Due to analysis in different laboratory facilities and scarcity of tissue *MSTN* qPCR was performed in 5 of these 11 volunteers and the other qPCRs as well as histologic investigations were performed in the other 6 volunteers (see Table 1).

**Table 1:** Baseline characteristics

**Baseline characteristics**	
*n*	83
Age (years)	53.0 [40.5/67.0]
Gender (m/f)	57/26 [68.7%/31.3%]
BMI (kg/m^2^)	27.1 [23.4/29.9]
ICU length of stay (days)	28.0 [20.0/42.0]
Time of first awakening (days after admission)	12.0 [9.0/20.0]
Survival (non-survivors/survivors)	14/69 [16.9%/83.1%]
**Catastrophic event leading to ICU admission**	
ARDS	28 [33.7%]
Sepsis	20 [24.1%]
Trauma	19 [22.9%]
CNS	15 [18.1%]
Miscellaneous	1 [1.2%]
**Illness severity at ICU admission**	
SOFA score	13 [10/15]
APACHE	23 [17/28]
SAPS2	52 [39/63]
**Time interval between ICU admission and muscle biopsy**	
N	59
Biopsy day (days after admission)	16.0 [14.0/19.5]
RASS	-3.0 [-4.0/-1.0]
Percent of days with RASS > − 3	40.0 [22.3/64.6]
Noradrenalin (µg/kg*min)	0.05 [0.03/0.10]
Noradrenalin days (days noradrenalin was required to maintain blood pressure)	9.0 [5.0/12.0]
Percent of days with septic shock (%)	21.4 [8.6/43.6]
**Intervention quantity**	
Net time patient received physiotherapy per day until muscle biopsy (minutes)^a^	18.2 [13.3/21.7]
Net time patient received physiotherapy per day until ICU discharge (minutes)^a^	20.0 [14.2/22.8]
**Healthy controls—plasma**	
*n*	91
Age (years)	53.0 [42.0/66.5]
Gender (m/f)	51/40 [56.0%/44.0%]
**Healthy controls—muscle biopsy specimen**	
*n*	11
Age (years)	58.0 [44.0/56.0]
Gender (m/f)	7/4 [63.6%/36.4%]

Values for metric variables are presented as median and interquartile range and for categorical variables as count and percentages

BMI, body mass index; ICU, intensive care unit; ARDS, acute respiratory distress syndrome; CNS, central nervous system; SOFA, Sepsis-related Organ Failure Assessment score; SAPS2, Simplified Acute Physiology Score; RASS, Richmond Agitation Sedation Scale

^a^Time shown is the time the patient received the actual physiotherapeutic intervention during which the muscle was stimulated not including preparation or documentation

### Intervention

Patients from the observational studies received standard physiotherapy (sPT) as prescribed by the treating physician. These patients were compared to those in the prospective interventional study that received protocol-based physiotherapy guided by daily mobilization goals that were defined during a multiprofessional ward round. The daily mobilization goals were provided by a stepwise approach starting at level 1 (no mobilization) until level 5 (intensified therapy with activities of daily living). Randomization allocated patients to just protocol-based physiotherapy (pPT) or protocol-based physiotherapy and additional muscle activating measures (pPT + adMeas), meaning neuromuscular electrical stimulation and/or whole-body vibration, daily for 20 min per additional measure, leading to three different groups (standard physiotherapy (sPT), protocol-based physiotherapy (pPT) and protocol-based physiotherapy + muscle activating measures (pPT + adMeas)) that were compared. Further details have been published [26].

### Procedures

All patients underwent surgical muscle biopsy of the M. vastus lateralis 15 days (respectively closest possible day) after ICU admission (Table 1).

Determination of expression levels for *MYH1, MYH2, MYH4, CAPN1, CASP3, TRIM62, TRIM63, FBXO32, PSMB2, IL6, TNFalpha and SAA1/2* as well as histologic analysis were performed as described previously [26]. Gene expression for *MSTN* was performed as outlined in the Additional file 1.

Blood samples were collected from ICU patients on day 4, 8 and 14 (or closest possible day) after ICU admission for determination of myostatin plasma levels. Myostatin plasma concentrations were measured using commercial ELISA (GDF-8/Myostatin Immunoassay; Catalog Number DGDF80, R&D Systems, Minneapolis, USA) (inter-assay CV 3.1–6.0%, intra-assay CV 1.8–5.4%).

Muscle strength was assessed using the Medical Research Council (MRC) score at first adequate awakening and at ICU discharge according to the criteria established by DeJonghe et al. [29]. Diagnosis of ICUAW was confirmed when an average score below 4 across all tested muscles was observed [1].

Electrophysiological evaluations were performed by an experienced neurologist after the first week in the ICU via a portable two-channel Keypoint Medtronic equipment (Skovlunde, Denmark). Patients were categorized into CIM if compound muscle action potential after direct muscle stimulation showed an amplitude below 3.0 mA and CIP when sensory nerve action potential showed a reduced amplitude. If both criteria were fulfilled, patients were categorized into CIM/CIP. Further details have been published [30].

Hyperinsulinemic–euglycemic clamp to determine the insulin sensitivity index was performed as described previously [11].

### Subgroup analysis

Patients were categorized according to myostatin plasma levels on day 14 into two subgroups: according to increased/decreased levels when compared to healthy controls.

### Statistics

Categorical variables are shown as counts and percentages, while metric variables are shown as median with interquartile range (IQR). Values for mRNA expression and plasma concentrations were normalized to healthy controls and are shown as fold change. Statistical difference was tested accordingly with a Mann–Whitney U and Kruskal–Wallis test for metric variables and between subject differences, a general linear model for repeated measures for metric variables and within subject differences and a chi-square test for categorical variables. Post hoc comparison of different groups within the general linear model for repeated measures was made applying Bonferroni correction for multiple comparison. Correlation analyses were performed with the Spearman’s rank correlation coefficient. A priori a two-tailed alpha level of < 0.05 was defined to indicate statistical significance. Statistical analysis was performed with SPSS (IBM Corp. SPSS Statistics for Macintosh, Version 26.0. Armonk, NY, USA). Graphs were designed with GraphPad Prism (Version 7.00 for Macintosh, GraphPad Software, La Jolla California USA).

## Results

Eighty-three patients (33 from the observational study and 50 from the interventional trial) were included into the study (see Additional file 1: Fig. S1). Baseline characteristics for all patients are shown in Table 1.

### Myostatin trajectory

**Fig. 1:**
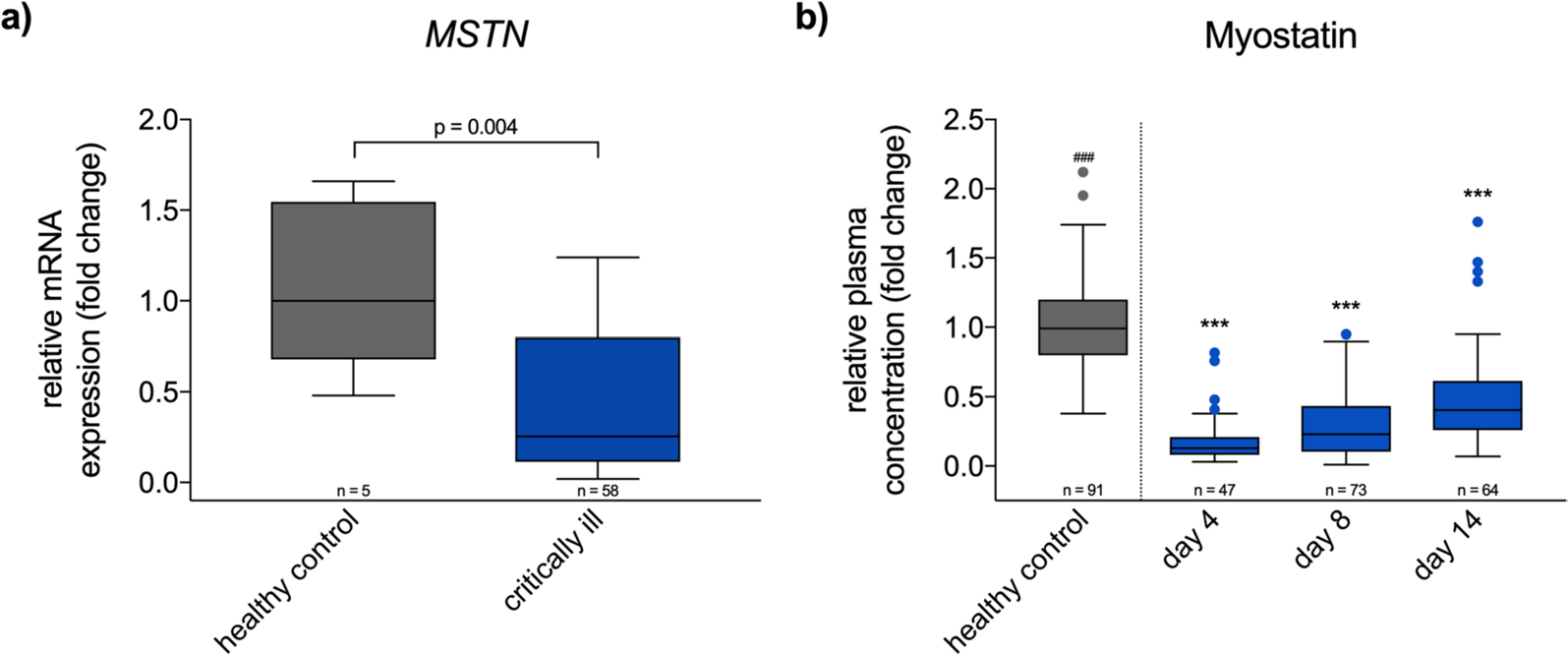
*MSTN* gene expression on day 15 and myostatin plasma trajectory. **a** MSTN gene expression was significantly decreased in critically ill patients (median [IQR] fold change: 1.00 [0.68–1.54] vs. 0.26 [0.11–0.80]; *p* = 0.004). **b** Myostatin plasma concentration over time showed significantly decreased values in critically ill patients. A recovery throughout the first 14 days can be observed (GLM median [IQR] fold change: healthy control vs. day 4 vs. day 8 vs. day 14—median [IQR] 0.99 [0.80–1.20] vs. 0.13 [0.08–0.21] vs. 0.23 [0.10–0.44] vs. 0.40 [0.26–0.61]; *p* < 0.001; *n* = 36 patients with values from all three timepoints were analyzed). GLM = general linear model for the factor “time” in critically ill; mRNA = messenger ribonucleic acid. ^###^*p* < 0.001 for Kruskal–Wallis test between healthy controls and critically ill; ***p < 0.001 for post hoc test comparison with healthy controls

Critically ill patients showed a significantly reduced *MSTN* gene expression in skeletal muscle when compared to healthy controls (*p* = 0.004; Fig. 1a). Reduced myostatin plasma levels were observed during the first 2 weeks of the ICU stay (*p* < 0.001), and were pronounced during the early phase of ICU treatment but with a significant increase over time (*p* < 0.001; Fig. 1b; see Additional file 1: Table S1 for absolute values). Patients with plasma myostatin levels on day 14 above the levels of healthy controls were significantly younger (see Additional file 1: Table S2).

### Muscle strength and physical function

**Fig. 2:**
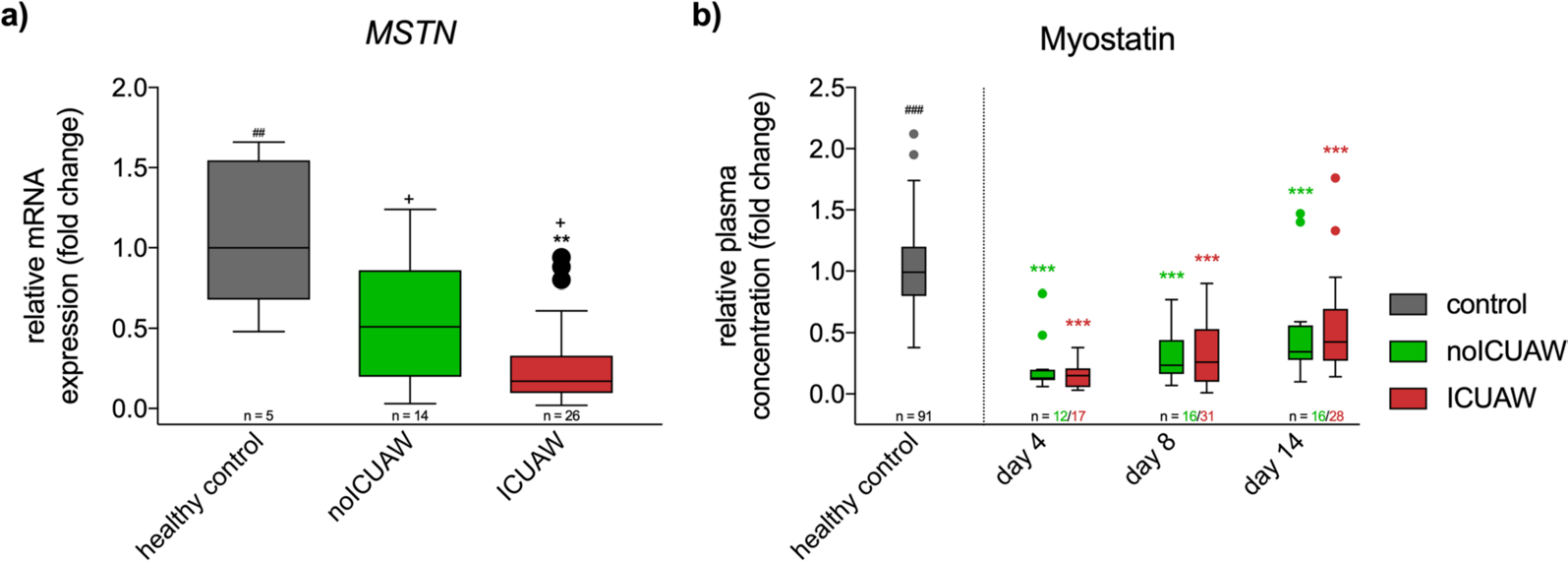
Differences in MSTN gene expression on day 15 and myostatin plasma trajectory in patients diagnosed with ICUAW at ICU discharge. **a** MSTN gene expression was significantly decreased in all critically ill patients independent of the ICUAW diagnosis. Patients with ICUAW presented furthermore a significant reduction in MSTN gene expression over those without ICUAW. **b** Myostatin plasma trajectory shows significantly decreased values that recover over time independent of ICUAW, while no differences between patients with and without ICUAW can be observed (GLM: *p* < 0.001; *n* = 9 patients with ICUAW and *n* = 15 without ICUAW with values from all three timepoints were analyzed). GLM = general linear model for the factor “time” in critically ill; mRNA = messenger ribonucleic acid. ^###^*p* < 0.001 for Kruskal–Wallis test between healthy controls and critically ill; **p < 0.01 and ***p < 0.001 for post hoc test comparison with healthy controls; ^+^*p* < 0.05 for post hoc test comparison between critically ill

Muscular *MSTN* gene expression on day 15 after ICU admission was significantly lower in ICUAW patients compared to controls and patients without ICUAW, while no difference was detected between controls and patients without ICUAW (Fig. 2a). Patients with and without ICUAW at ICU discharge had significantly decreased myostatin plasma levels, which significantly increased over time. A difference between patients with ICUAW and patients without ICUAW was not observed (Fig. 2b). Low myostatin plasma concentrations on day 8 showed a correlation to reduced muscle strength at first awakening, while no correlation was observed at ICU discharge (correlation coefficient 0.339; R^2^ = 0.109; *p* = 0.020; see Additional file 1: Fig. S2, Table S3).

### Interventions

**Fig. 3:**
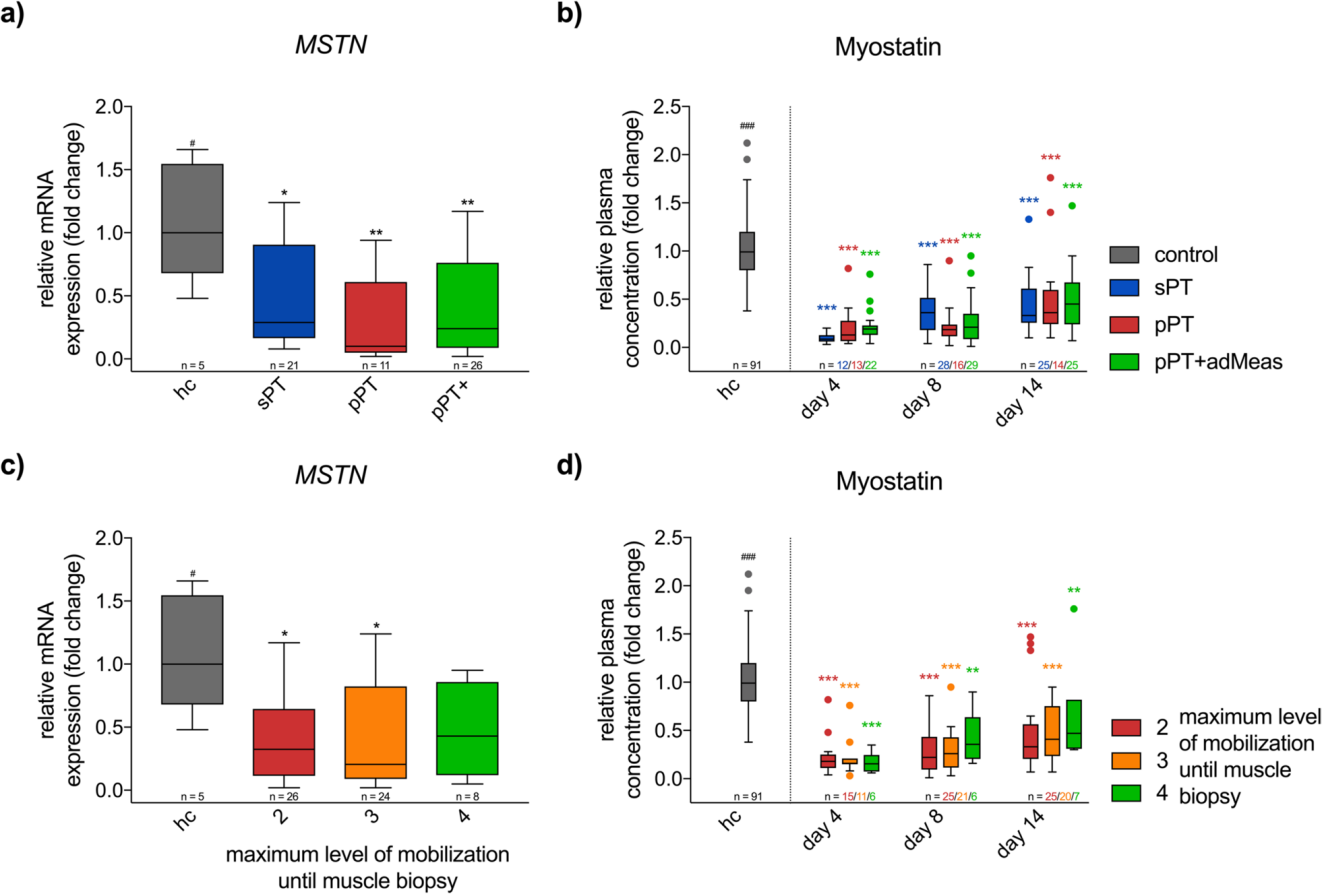
Impact of maximum level of mobilization as well as different physiotherapeutic regimens on MSTN gene expression on day 15 and myostatin plasma trajectory. **a** MSTN gene expression was not influenced by standard physiotherapy (sPT), protocol-based physiotherapy (pPT) or protocol-based physiotherapy with additional muscle activating measures (pPT +) as it was significantly decreased over healthy controls (hc) in all groups. **b** Myostatin plasma levels showed a similar pattern with decreased values in all critically ill patients independent of the intervention and with a significant recovery of time (GLM: *p* < 0.001; *n* = 7 patients receiving sPT, *n* = 10 receiving pPT and *n* = 19 receiving pPT + adMeas with values from all three timepoints were analyzed). **c** MSTN gene expression did not show any difference due to the achieved level of mobilization and neither a reduction over baseline values. **d** Myostatin plasma trajectory presented similarly without any impact of the achieved level of mobilization but a significant reduction in all critically ill patients. A significant recovery over time was also evident (GLM: *p* = 0.001; *n* = 14 patients reaching level 2, *n* = 10 reaching level 3 and n = 5 reaching level 4 with values from all three timepoints were analyzed). GLM = general linear model for the factor “time” in critically ill; mRNA = messenger ribonucleic acid. ^#^*p* < 0.050, ^##^*p* < 0.010 and ^###^*p* < 0.001 for Kruskal–Wallis test between healthy controls and critically ill. *p < 0.05, **p < 0.01 and ***p < 0.001 for post hoc test comparison with healthy controls

Compared to healthy controls, *MSTN* gene expression showed a significant reduction in all critically ill patients regardless of the intervention they were randomized to or the level of mobilization they achieved until muscle biopsy (Fig. 3a, c). Myostatin plasma levels presented a similar pattern for the interventions as well as maximum level of mobilization with a significant reduction in all critically ill patients and a significant recovery over time with no between-group differences (Fig. 3b, d).

### Electrophysiology

*MSTN* gene expression was decreased in all critically ill patients irrespective of the electrophysiological classification (except in patients with CIP; n.s.). No difference in expression levels between these groups could be observed (see Additional file 1: Fig. S3a). Similarly, myostatin plasma levels were significantly reduced at all timepoints regardless of the electrophysiological classification (see Additional file 1: Fig. S3b). No correlation between compound muscle action potential after direct muscle stimulation (dmCMAP) and neither *MSTN* gene expression nor myostatin plasma levels were observed (see Additional file 1: Table S4). No patient with myostatin plasma levels higher than healthy controls on day 14 was classified as CIP, CIM or CIP/CIM (see Additional file 1: Fig. S3c).

### Muscle homeostasis

Critically ill patients show significantly increased expression levels of genes related to muscle atrophy and inflammation when compared to healthy controls. Myosin protein content and myocyte cross-sectional area were not different according to the myostatin groups (see Additional file 1: Fig. S4a,b).

### Insulin sensitivity

Critically ill patients with myostatin plasma levels higher than healthy controls also showed a significantly higher insulin sensitivity index (see Additional file 1: Fig. S5a). In line with that, a direct correlation between the insulin sensitivity index and myostatin plasma levels on day 14 was observed (see Additional file 1: Fig. S5b, Table S5).

## Discussion

Muscular *MSTN* gene expression and myostatin plasma levels presented a congruent decrease in critically ill patients that increased over time while not reaching baseline values 14 days after ICU admission. *MSTN* gene expression was significantly lower in patients with clinical weakness. Otherwise, no difference was observed when stratifying patients according to weakness or electrophysiological classification. Markers of muscle atrophy were not associated with myostatin plasma levels. The association of myostatin plasma levels and insulin resistance was contrary to our hypothesis with decreased values in patient with more severe insulin resistance. No effect of early mobilization or additional muscle activating measure on neither myostatin plasma levels nor *MSTN* gene expression could be observed.

Previous studies indicate interest in therapeutic modulation of myostatin as an established regulator of muscle mass. It was hypothesized that inhibition of myostatin could ameliorate muscle atrophy and improve muscle function [14, 31]. Multiple therapeutic options such as myostatin-binding proteins or myostatin-neutralizing antibodies have been developed and tested [32, 33]. We have therefore included plasma values into our study to be able to establish a rationale for the application of these inhibitors in ICUAW and to identify a potential biomarker. However, published results in hereditary muscle disease were rather disappointing and previous trials had to be discontinued partly due to adverse events and lack of efficacy (NCT02310763, NCT02907619) [34].

Contrary to the substantially increased muscle mass in animals and humans with genetic alterations leading to lacking myostatin effect did anti-myostatin therapy in hereditary muscle disease not lead to an increase in muscle mass [35]. Mariot et al. investigated the causes for these diverging observations [35]. The authors showed that patients with Duchenne muscular dystrophy as well as spinal muscular atrophy have reduced levels of both systemic myostatin and activin (also a negative regulator of muscle mass from the TGF-beta superfamily) while typically having increased levels of circulating follistatin as an important inhibitor of myostatin leading to muscle growth [35, 36]. Moreover, they found that the same patients with Duchenne muscular dystrophy also have reduced myostatin expression levels in skeletal muscle [35]. Furthermore, patients with inclusion body myositis show not only reduced myostatin expression levels in skeletal muscle but also an increased follistatin expression [35]. These findings are in line with our data from critically ill patients, data from a murine sepsis model from Smith et al., and the findings presented by Burch et al. in patients with genetic neuromuscular disorders [37–39]. Additionally, Puthucheary et al. identified no change over time in *MSTN* gene expression in critically ill patients between ICU day 1 and 7, while it remains unclear if the values were reduced due to a lacking comparison with healthy controls [8]. These findings diverge from ours as the group did not observe recovery over time, which might be an effect of sample size as Wirtz et al. also observed significantly reduced myostatin plasma levels [39]. In agreement with the data and interpretation of Mariot et al., we hypothesize that the myostatin downregulation could be a compensatory mechanism to muscle atrophy induced by the underlying muscular disease [35]. These findings are underlined by the fact that Mariot et al. observed an upregulation of follistatin, which has an antagonistic effect on myostatin and leads to muscle growth [35].

Interestingly, respective compensatory mechanisms could be hampered during critical illness as it may dependent on the insulin-like growth factor receptor. In their work, Kalista et al. observed that the hypertrophic effect of follistatin is mediated via the IGF-I receptor/Akt/mTOR pathway [40]. Previously, it was shown that the IGF-I receptor gene expression is significantly reduced in critically ill patients, especially those with CIM, which may blunt compensatory mechanisms [11]. Furthermore, we observed reduced mTOR protein levels in critically ill patients, which is also involved in the hypertrophic effect elicited by follistatin [11].

Myostatin increases the expression of MuRF1 and Atrogin-1 as key mediators of muscle atrophy and decreases the expression of myosin heavy chain [20]. Contrary to our hypothesis were MuRF1, Atrogin-1 and/or myosin heavy chains appeared not to be associated with myostatin. The recent literature demonstrated an early increase in muscle protein synthesis in critically ill patients measured via labeled amino acids. This might be a result of the compensatory downregulation of myostatin [10].

The compensatory downregulation could also extend beyond muscle atrophy into insulin signaling as insulin resistance is a common observation during critical illness and high myostatin levels have also been associated with insulin resistance [21, 22]. Evidence suggests that myostatin might mediate insulin resistance in a similar fashion that was observed during critical illness, namely decreased GLUT4 expression and translocation [11, 41]. The direct correlation observed between myostatin and the insulin sensitivity index in critically ill patients is inverse to the correlation observed in healthy adults and might be a product of compensatory downregulation in order to improve insulin sensitivity in the critically ill patients [21].

It appears that the TGF-beta pathway while not being regulated via myostatin plays a critical role in ICUAW. Bloch et al. have shown that GDF-15, a myokine from the TGF-beta family similar to myostatin, is upregulated during critical illness [42]. This observation underlines that the atrophy mechanism during ICUAW is different from COPD as both myostatin and GDF-15 were increased in COPD patients [43].

Exercise is a powerful stimulus to increase muscle mass and function [44]. It has been shown to be linked to myostatin as plasma, muscle protein and muscle expression levels significantly decreased with exercise [22, 45]. We hypothesized that early mobilization mediates its beneficial effects on muscle mass via myostatin. However, we did not observe any effects of the mobilization strategies tested, such as protocol-based physiotherapy with and without muscle activating measures, nor of different levels of mobilization during the ICU stay, for example whether the patients achieved ambulation or not, on myostatin plasma levels or gene expression.

Nonetheless, our study has certain limitations. First, driven by study design, pooled data (which were recorded in a prospective fashion) from two independent clinical studies were analyzed retrospectively. Secondly, all patients were recruited within one academic center, which might hamper external validity. Third, driven by the observational nature of our investigations, further investigations are required to elucidate potential mechanism behind the downregulation of myostatin. As this was out of the scope of the current analysis, it should be pursued in subsequent studies. Last, due to the complex nature of the study with molecular analyses from biological samples in critically ill patients we have some missing values in certain analyses, which limits external validity of the results.

## Conclusion

Data from two prospective clinical studies demonstrate that myostatin gene expression and systemic protein levels are significantly decreased in critically ill patients with ICUAW. We conclude from our data that myostatin is likely not a key driver of muscle weakness, muscle atrophy, insulin resistance and/or electrophysiological alterations observed during critical illness. Improving muscle quality via therapeutic inhibition of myostatin to prevent or treat muscle weakness, muscle atrophy and/or insulin resistance during critical illness does not seem to have a robust pathophysiological rationale.

## Supplementary Information

**Additional file 1:** Additional methods, figure and tables.

## Data Availability

The datasets used and/or analyzed during the current study are available from the corresponding author on reasonable request.

## Abbreviations

Akt: Protein kinase b
ARDS: Acute respiratory distress syndrome
*CAPN1*: Calpain-1
*CASP3*: Caspase-3
CIM: Critical illness myopathy
CIP: Critical illness polyneuropathy
CNS: Central nervous system
dmCMAP: Direct muscle stimulation compound muscle action potential
ELISA: Enzyme-linked immunosorbent assay
*FBXO32*: F-box protein 32
FoxO1: Forkhead box O1 (FoxO1)
GDF-8: Myostatin
GLUT4: Glucose transporter type 4
ICU: Intensive care unit
ICUAW: Intensive care unit-acquired weakness
IGF-I: Insulin-like growth factor I
*IL6*: Interleukin 6
IQR: Interquartile range
MRC: Medical Research Council score
mRNA: Messenger ribonucleic acid
*MSTN*: Myostatin gene
mTOR: Mammalian target of rapamycin
*MuRF-1*: Muscle RING-finger protein-1
*MYH1*: Myosin heavy chain 1
*MYH2*: Myosin heavy chain 2
*MYH4*: Myosin heavy chain 3
pPT: Protocol-based physiotherapy
pPT + adMeas: Protocol-based physiotherapy + additional muscle activating measures
*PSMB2*: Proteasome 20S Subunit Beta 2
qPCR: Quantitative polymerase chain reaction
RASS: Richmond agitation sedation
SOFA: Sepsis-related Organ Failure Assessment Score
sPT: Standard physiotherapy
TGF-beta: Transforming growth factor beta 1
*TNFalpha*: Tumor necrosis factor alpha
*TRIM62*: Tripartite motif-containing 62
*TRIM63*: Tripartite motif-containing 63
*SAA1/2*: Serum amyloid ½
