# Supplementary material for: Muscular myostatin gene expression and plasma concentrations are decreased in critically ill patients
Source: Crit Care. 2022 Aug 3;26:237. doi: 10.1186/s13054-022-04101-1 (PMC9347123; doi:10.1186/s13054-022-04101-1)
Supplement: Supplementary file 1 — Additional file 1: Additional methods, figure and tables. [file 13054_2022_4101_MOESM1_ESM.pdf]

Additional File 1

Muscular myostatin gene expression and plasma concentrations are decreased in critically ill patients

Julius J. Grunow, MD[1], Katja Reiher, MD[1], Niklas M. Carbon, MD[1], Lilian Jo Engelhardt, MD[1,2], Knut Mai, MD[3], Susanne Koch, MD[1], Joerg C. Schefold, MD[4], Werner Z'Graggen, MD[5], Stefan J. Schaller, MD[1,6], Jens Fielitz, MD[7,8,9], Joachim Spranger, MD[3], Steffen Weber-Carstens, MD[1], Tobias Wollersheim, MD[1]

Affiliations:

1. *Charité – Universitätsmedizin Berlin, corporate member of Freie Universität Berlin, Humboldt-Universität zu Berlin, Department of Anesthesiology and Operative Intensive Care Medicine (CCM/CVK), Augustenburger Platz 1, 13357 Berlin, Germany*
2. *Charité – Universitätsmedizin Berlin, corporate member of Freie Universität Berlin, Humboldt-Universität zu Berlin, Institute of Medical Informatics, Charitéplatz 1, 10117 Berlin, Germany.*
3. *Charité – Universitätsmedizin Berlin, corporate member of Freie Universität Berlin, Humboldt-Universität zu Berlin, Department of Endocrinology and Metabolic Diseases, Charitéplatz 1, 10117 Berlin, Germany*
4. *University Bern, Bern University Hospital, Inselspital, Department of Intensive Care Medicine, Freiburgstrasse 18, 3010 Bern, Switzerland*
5. *University Bern, Bern University Hospital, Inselspital, Departments of Neurology and Neurosurgery, Freiburgstrasse 18, 3010 Bern, Switzerland*
6. Technical University of Munich, School of Medicine, Department of Anesthesiology and Intensive Care, Ismaninger Straße 22, 81675 Munich, Germany
7. *Charité – Universitätsmedizin Berlin, Max Delbrück Center (MDC) for Molecular Medicine in the Helmholtz Association, Experimental and Clinical Research Center (ECRC), Lindenberger Weg 80, 13125 Berlin, Germany*
8. *University Medicine Greifswald, Department of Internal Medicine B, Cardiology, Greifswald, Germany*
9. DZHK (German Center for Cardiovascular Research), partner site Greifswald, Greifswald, Germany

Corresponding author:

Prof. Dr. med. Steffen Weber-Carstens

Department of Anesthesiology and Operative Intensive Care Medicine (CCM, CVK)

Charité - Universitätsmedizin Berlin, Germany

Augustenburger Platz 1

13353 Berlin

Germany

e-mail: steffen.weber-carstens@charite.de

tel: +49 (0)30 450 651 055

ORCID: 0000-0001-7405-0172

Methods

MSTN expression levels frozen tissue was homogenized and total RNA was isolated according to the manufacturer's instructions of Trizol Reagent (Invitrogen, CA, USA). RNA samples were stored at −80 °C until assayed. Complementary DNA (cDNA) synthesis was done according to manufacture manual (High Capacity RNA-tocDNA Kit; Applied Biosystems, Foster City, CA). Samples were analyzed in triplicate with Power SYBR Green PCR Master Mix (Applied Biosystems). Real-time quantitative polymerase chain reaction was performed using an ABI PRISM 7300 System (using SDS 1.4 system software, Applied Biosystems). The expression level of cyclophyllin A was used as an internal control. The used primer sequences were: cyclophyllin A (forward: TGTGAAGTCACCACCCTGACACAT; reverse: AGACAAGGTCCCAAAGACAGCAGA) and myostatin (forward GGCTCAAACAACCTGAATCC; reverse: TCCCTTCTGGATCTTTTTGG; Life technologies). Cycle threshold values were used to calculate the amount of amplified polymerase chain reaction product in comparison to the housekeeping gene cyclophyllin A. The relative amounts of each transcript were analyzed using the 2−ΔC(t) method [1].

References

1. Mai K, Klug L, Rakova N, Piper SK, Mahler A, Bobbert T, Schulz-Menger J, Spranger J, Boschmann M, Luft FC: **Hypoxia and exercise interactions on skeletal muscle insulin sensitivity in obese subjects with metabolic syndrome: results of a randomized controlled trial**. *Int J Obes (Lond)* 2020, **44**(5):1119-1128.

Figures

Figure A1 Inclusion flow chart

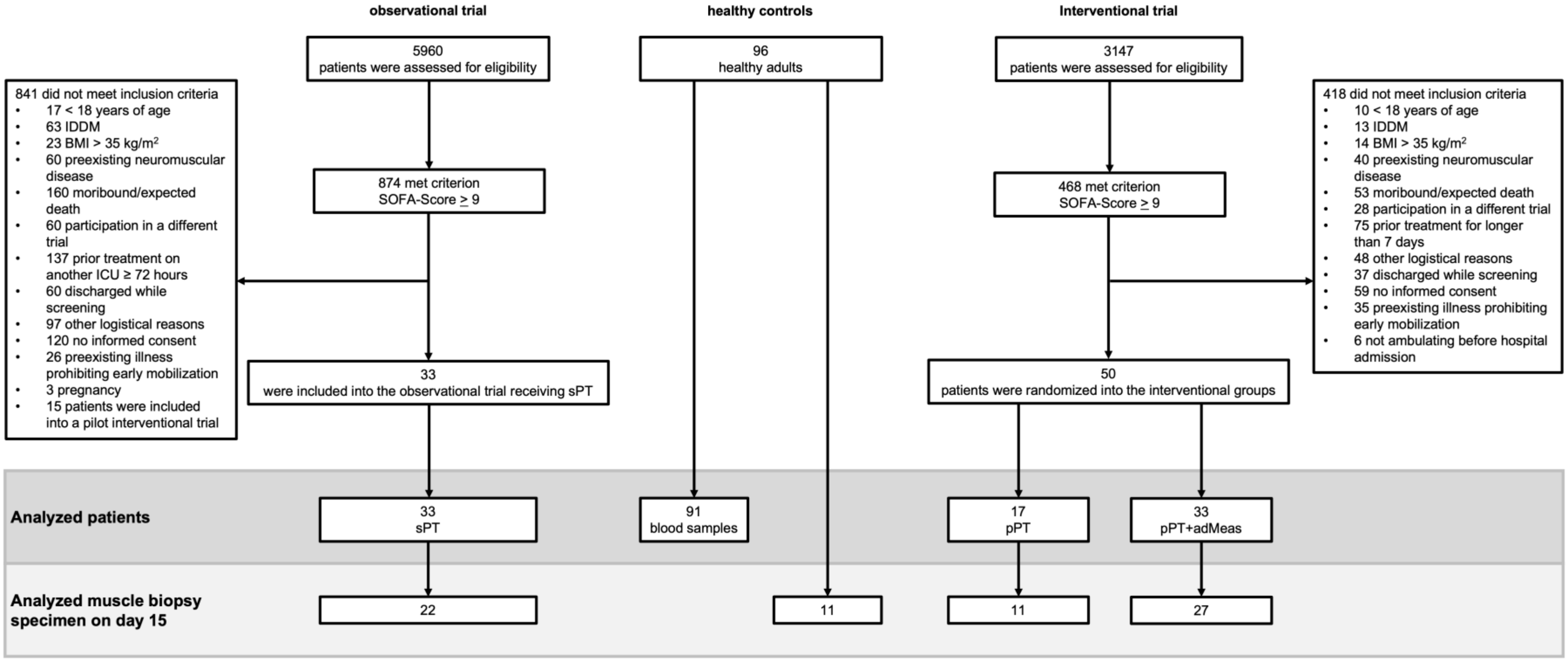

Inclusion flow chart depicting included and excluded respectively enrolled and analyzed patients. IDDM = insulin dependent diabetes mellitus; ICU = Intensive Care Unit; SOFA-Score = Sepsis-related Organ Failure Assessment score; sPT = standard phyiotherapy; pPT = protocol-based physiotherapy; pPT+adMeas = protocol-based physiotherapy + additional muscle activating measures

Figure A2 Correlation between myostatin plasma levels on day 8 and muscle strength measured via Medical Research Council score at first awakening

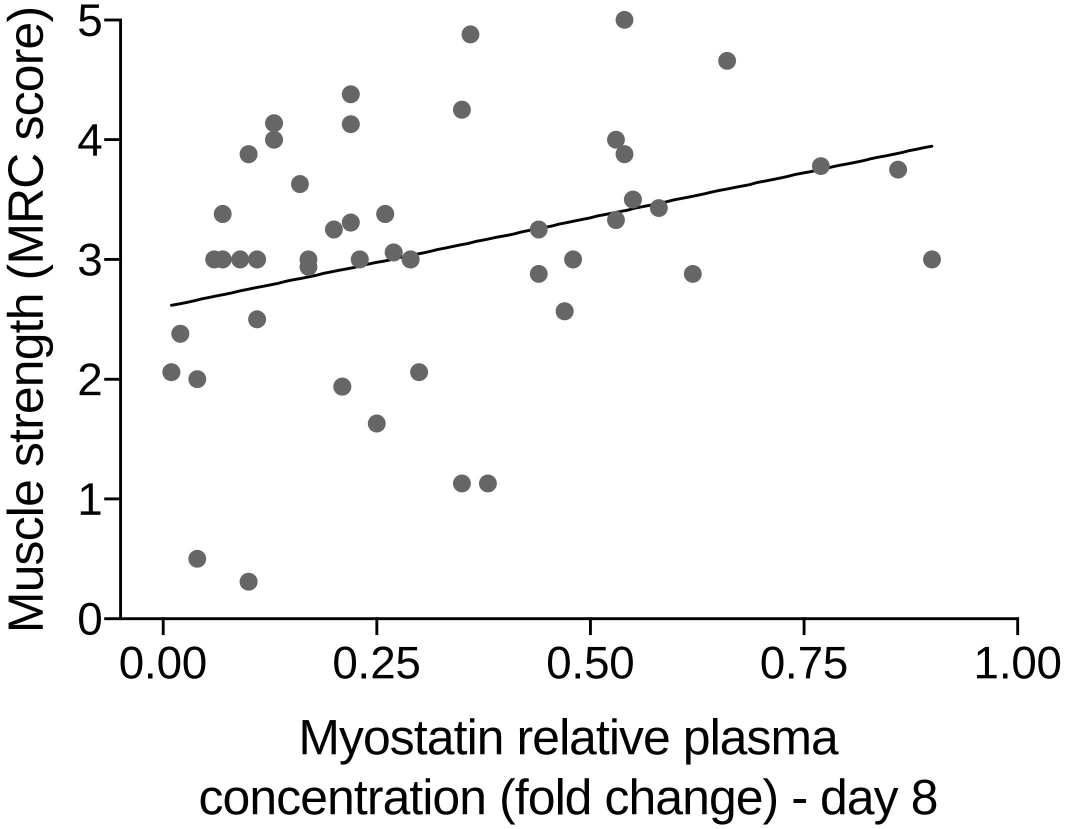

Myostatin plasma levels on day 8 show a significant positive correlation to muscle strength measured via MRC score at first adequate awakening.

MRC = Medical Research Council Score

See Table A3 for correlation coefficient and significance value.

Figure A3 Differences in MSTN gene expression and myostatin plasma trajectory in patients according to electrophysiological diagnosis

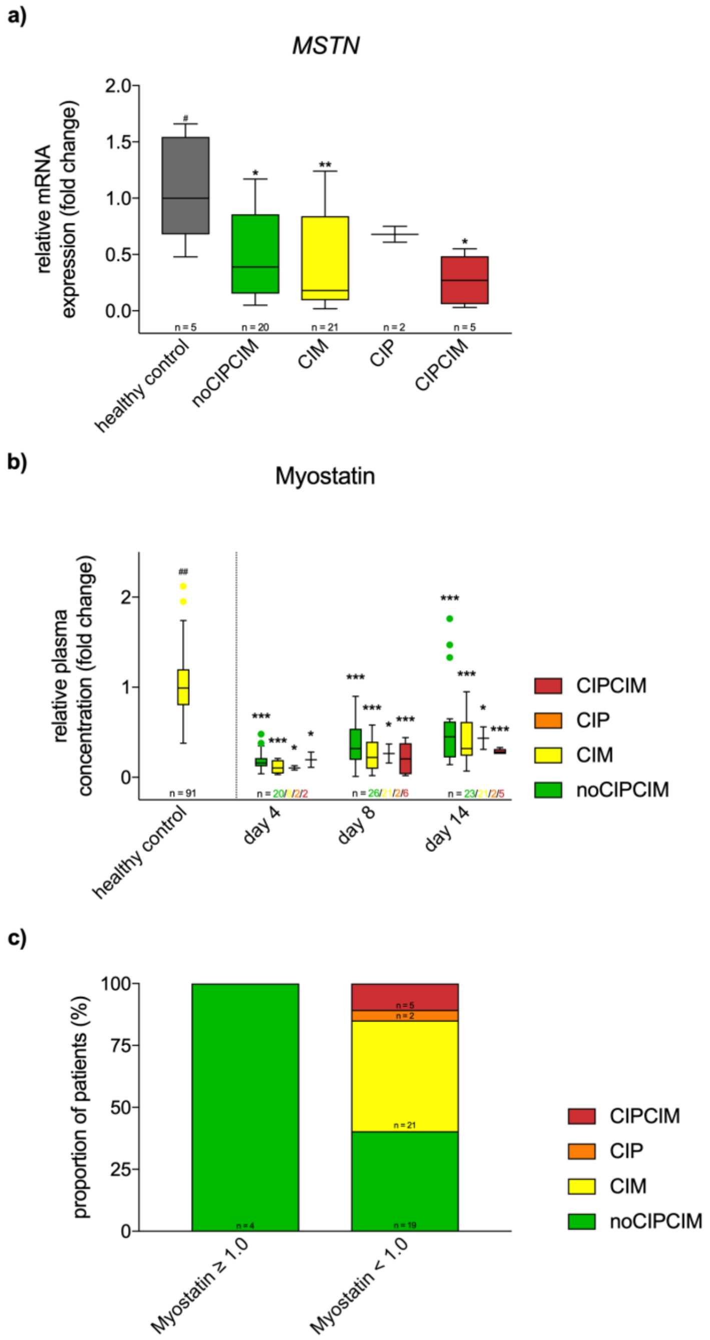

a) MSTN gene expression was significantly lower in all critically ill patients except those diagnosed with CIP.

b) Myostatin plasma concentration was significantly lower in all critically ill patients irrespective of the electrophysiological diagnosis but the recovery over time did not reach statistical significance [GLM: p = 0.106; n = 15 patients classified as noCIPCIM, n = 8 patients classified as CIM, n = 2 patients classified as CIP and n = 2 patients classified as CIPCIM with values from all three timepoints were analyzed]

c) Patients with myostatin plasma levels on day 14 at or above healthy controls did not show any signs of CIP, CIM or CIPCIM in their electrophysiological evaluation.

GLM = general linear model for the factor "time" in critically ill; mRNA = messenger ribonucleic acid; CIM = Critical Illness Myopathy; CIP = Critical Illness Polyneuropathy; CIPCIM = Critical Illness Polyneuropathy and Myopathy

##: $p < 0.010$ for Kruskal-Wallis test between healthy controls and critically ill

**: $p < 0.05$ and ***: $p < 0.001$ for post-hoc test comparison to healthy controls*

Figure A4 Molecular parameters of muscle homeostasis according to myostatin cluster

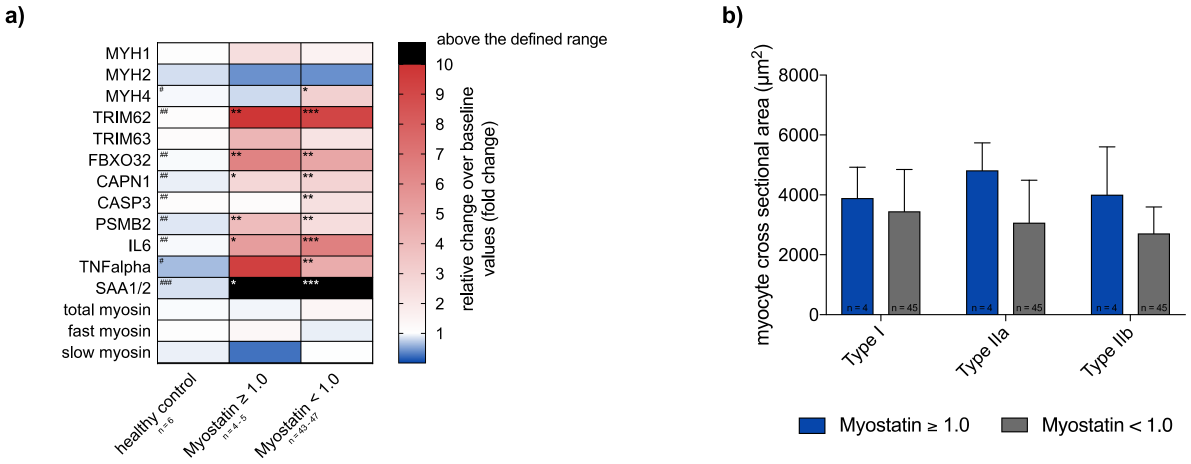

a) All critically ill patients show a typical pattern of molecular parameters with upregulation of muscle protein degradation and inflammation. No difference between the two clusters of Myostatin plasma levels were present.

b) Myocyte cross sectional area also showed no difference between the two Myostatin groups.

#: $p < 0.050$, ##: $p < 0.010$ and ###: $p < 0.001$ for Kruskal-Wallis test between healthy controls and critically ill

**: $p < 0.05$, **: $p < 0.01$ and ***: $p < 0.001$ for post-hoc test comparison to healthy controls*

Figure A5 Insulin Sensitivity Index according to myostatin cluster and between myostatin plasma level on day 14 and Insulin Sensitivity Index

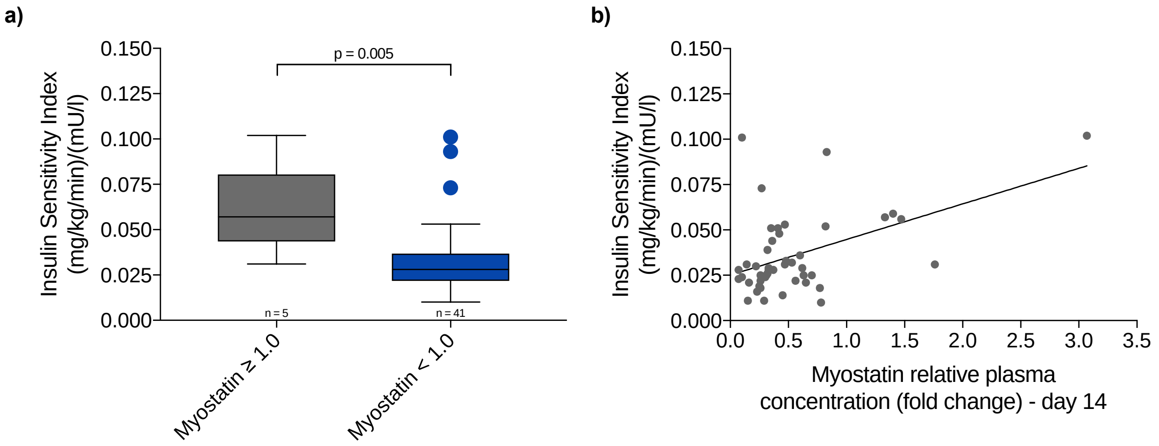

a) Critically ill patients with plasma Myostatin levels at or above healthy controls show a significantly higher Insulin Sensitivity Index.

b) Myostatin plasma levels on day 14 show a significant positive correlation to the Insulin Sensitivity Index.

See Table A5 for correlation coefficient and significance value.

Tables

Myostatin plasma values (pg/ml)	Median	Interquartile Range
Healthy Controls	2990.3	2417.0 – 3609.9
Day 4	406.4	233.1 - 632.7
Day 8	707.3	314.8 - 1312.8
Day 14	1213.9	790.3 – 1842.5

Table A1 Myostatin plasma values

Values for metric variables are presented as median and interquartile range.

**Baseline characteristics**	Myostatin ≥ 1.0	Myostatin < 1.0	p-value
n	6	58	
Age (years)	25.0 [21.0/34.0]	55.5 [44.0/68.0]	< 0.001
Gender (m/f)	5/1 [83.3%/16.7%]	38/20 [65.5%/34.5%]	0.376
BMI (kg/m^2)	23.7 [22.9/27.8]	27.0 [23.4/30.0]	0.261
ICU length of stay (days)	39.5 [29.0/43.0]	31.0 [21.0/43.0]	0.521
Time of first awakening (days after admission)	23.0 [19.0/23.0]	12.0 [9.0/20.0]	0.179
Survival (non-survivors/survivors)	0/6 [0.0%/100.0%]	6/52 [10.3%/89.7%]	0.408
**Catastrophic event leading to ICU admission**			
ARDS	2 [33.3%]	19 [32.8%]	0.153
Sepsis	0 [0.0%]	13 [22.4%]	
Trauma	4 [66.7%]	13 [22.4%]	
CNS	0 [0.0%]	12 [20.7%]	
Miscellaneous	0 [0.0%]	1 [1.7%]	
**Illness severity at ICU admission**			
SOFA score	14.0 [13.0/17.0]	12.0 [10.0/14.0]	0.160
APACHE	16.5 [14.0/27.0]	23.0 [17.0/28.0]	0.233
SAPS2	41.0 [33.0/65.0]	52.0 [39.0/63.0]	0.410
**Time interval between ICU admission and muscle biopsy**			
n	5	47	
Biopsy day (days after admission)	13.0 [12.0/15.0]	16.0 [14.0/19.5]	0.210
RASS	-3.0 [-4.0/-1.0]	-3.0 [-4.0/-1.0]	0.928
Percent of days with RASS > - 3	40.0 [40.0/54.5]	40.0 [15.0/64.6]	0.431
Noradrenalin (µg/kg*min)	0.05 [0.01/0.10]	0.05 [0.02/0.10]	0.651
Noradrenalin days (days noradrenalin was required to maintain blood pressure)	4.0 [4.0/11.0]	10.0 [6.0/12.0]	0.210
Percent of days with septic shock (%)	33.3 [18.2/40.0]	18.2 [8.6/45.6]	0.566
**Intervention quantity**			
Net time patient received physiotherapy per day until muscle biopsy (minutes)[+]	16.1 [14.1/20.4]	17.4 [12.1/22.3]	0.904
Net time patient received physiotherapy per day until ICU discharge (minutes)[+]	19.3 [12.1/29.1]	20.1 [15.2/22.9]	0.902

Table A2 Baseline characteristics for myostatin groups

Values for metric variables are presented as median and interquartile range and for categorical variables as count and percentages. Mann-Whitney U or Chi-Square Test were used to calculate statistical significance. BMI = Body Mass Index; ICU = Intensive Care Unit; ARDS = Acute Respiratory Distress Syndrome; CNS = Central Nervous System SOFA = Sepsis-related Organ Failure Assessment score; SAPS2 = Simplified Acute Physiology Score; RASS = Richmond Agitation Sedation Scale; +time shown is the time the patient received the actual physiotherapeutic intervention during which the muscle was stimulated not including preparation or documentation.

	MRC score first awakening			MRC score ICU discharge		
	Correlation coefficient	R^2	p	Correlation coefficient	R^2	p
MSTN – relative mRNA expression (fold change)	- 0.089	0.001	0.586	0.212	0.014	0.189
Myostatin day 4 – relative serum concentration (fold change)	0.025	0.010	0.897	- 0.063	0.002	0.747
Myostatin day 8 – relative serum concentration (fold change)	0.339	0.109	0.020	0.169	0.058	0.256
Myostatin day 14 – relative serum concentration (fold change)	0.161	0.088	0.298	0.044	0.042	0.776

Table A3 Correlation MSTN, myostatin and muscle strength

Correlations were assessed with Spearman's rank correlation coefficient.

mRNA = messenger ribonucleic acid.

	dmCMAP		
	Correlation coefficient	R^2	p
MSTN – relative mRNA expression (fold change)	0.142	<0.0001	0.358
Myostatin day 4 – relative serum concentration (fold change)	0.156	0.049	0.411
Myostatin day 8 – relative serum concentration (fold change)	- 0.043	<0.0001	0.764
Myostatin day 14 – relative serum concentration (fold change)	0.135	0.002	0.366

Table A4 Correlation MSTN, myostatin and direct muscle stimulation compound muscle action potential

Correlations were assessed with Spearman's rank correlation coefficient. dmCMAP = direct muscle stimulation compound muscle action potential; mRNA = messenger ribonucleic acid.

	Insulin Sensitivity Index (mg/kg/min)/(mU/l)		
	Correlation coefficient	R^2	p
MSTN – relative mRNA expression (fold change)	-0.030	0.071	0.847
Myostatin day 4 – relative serum concentration (fold change)	0.199	0.077	0.341
Myostatin day 8 – relative serum concentration (fold change)	0.112	0.021	0.463
Myostatin day 14 – relative serum concentration (fold change)	0.357	0.228	0.015

Table A5 Correlation MSTN, myostatin and Insulin Sensitivity Index

Correlations were assessed with Spearman's rank correlation coefficient.

mRNA = messenger ribonucleic acid.
